# Polypropylene vs. stainless-steel wire suture: short-term recurrence rate after shouldice primary inguinal hernia repair, a non-inferior analysis among 1120 patients. A case–control study

**DOI:** 10.1007/s10029-024-03110-z

**Published:** 2024-08-29

**Authors:** Christoph Paasch, Marguerite Mainprize, Richard Hunger, Fernando A C Spencer Netto

**Affiliations:** 1 University Hospital Brandenburg an Der Havel, Brandenburg an Der Havel, Brandenburg, Germany; 2 Shouldice Hospital, 7750 Bayview Ave, Thornhill, ON Canada; 3 Hamad General Hospital, Hamad Medical Corporation, Doha, Qatar

**Keywords:** Reoperation, Hernia recurrence, Shouldice repair, Stainless steel, Suture, Polypropylene

## Abstract

**Introduction:**

Polypropylene material is commonly used for posterior wall reconstruction in hernia repair, in contrast with the classically described stainless-steel wire used at Shouldice Hospital. This study was conducted to evaluate possible differences in Shouldice Repair outcomes using polypropylene or stainless-steel wire sutures.

**Methods:**

A prospective follow-up of consecutive patients who underwent elective unilateral Shouldice primary inguinal hernia repair at Shouldice Hospital between December 6, 2021, and September 1, 2022, was conducted. Data was collected from follow-up telephone calls as well as manually reviewing patient's charts. The primary objective was to determine whether the use of polypropylene was non-inferior to the use of stainless-steel wire, regarding the recurrence rate reported by the patients with a minimum follow-up of 1 year after Shouldice primary inguinal hernia repair.

**Results:**

A total of 1120 patients were contacted by telephone (polypropylene: 560; stainless-steel wire: 560). The median follow-up period was 16 months (interquartile range: 15–18). In 22 (1.96%) cases a surgical site infection was diagnosed. There was a total of 18 recurrences reported by the patients (1.6%). There was no statistical difference between the groups (polypropylene: 7 (1.25%) vs. stainless steel wire: 11 (1.96%), p > 0.05) for the recurrence rate.

**Conclusion:**

The use of polypropylene is non-inferior to the use of stainless-steel wire regarding recurrence rate at a median follow-up period of 16 months after elective unilateral Shouldice primary inguinal hernia repair. This finding may encourage other centers where stainless-steel wire is not easily available to perform the Shouldice Repair.

**Supplementary Information:**

The online version contains supplementary material available at 10.1007/s10029-024-03110-z.

## Introduction

The Shouldice Repair is a well-known and documented surgical technique for inguinal hernia repair [1, 2]. This repair was created and perfected by Dr. E.E. Shouldice and others between 1936 and the 1950s [3]. The classic component of a Shouldice Repair is the four-layer suturing of the tissue. This suturing was originally done using silk, but to improve strength and reduce infection, stainless-steel wire was implemented [3, 4].

Outside of Shouldice Hospital, polypropylene thread is commonly used for fascial closure and hernia repair, and this might be because polypropylene is easy to obtain and has proven to be an adequate suture material (monofilament with good handling, strength and casting) [5]. There is some learning curve associated with the stainless-steel wire, to avoid kinking and breaking [6]. The use of stainless-steel wire for Shouldice Repair was evaluated in 1995 by Hay et al., who conducted a prospective randomized clinical trial comparing Shouldice Repair with polypropylene (n = 419) and stainless-steel wire (n = 401) [7]. They compared only male patients and showed no statistical difference regarding recurrences in the 3 initial years after primary inguinal hernia repair (polypropylene 4.3% vs. stainless-steel wire 3.0%) [7]. This study aims to include more patients of all genders and provide results with current classifications for postoperative morbidity, with an adequate sample size to test non-inferiority.

In recent years, polypropylene sutures have also been used at Shouldice Hospital as an alternative to reconstruct the posterior wall of the inguinal canal. The purpose of this study was to further evaluate Shouldice Repair using polypropylene material and stainless-steel wire sutures.

## Methods

### Ethics

The project was approved by the Lakeridge Ethics Board (2023–05) and performed in accordance with the declaration of Helsinki.

### Study design

**Fig. 1:**
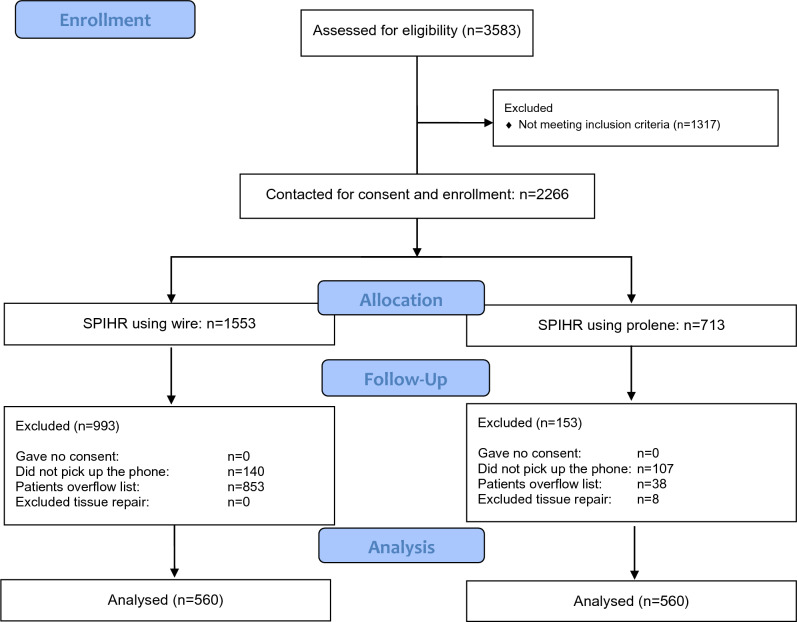
Flow chart diagram

A prospective follow-up of consecutive patients who underwent Shouldice primary inguinal hernia repair (SPIHR) at Shouldice Hospital between December 6, 2021, and September 1, 2022, was conducted. Data was collected from a follow-up telephone call conducted in September 2023, as well as manually reviewing patient operative notes and charts to collect biometric and perioperative data. We aimed to analyze 1120 (560 polypropylene and 560 stainless-steel wire with 0% decline to participate) to 1400 (700 polypropylene and 700 stainless-steel wire with 20% decline to participate) patients. A list of patients was provided for data review, information was gathered on patients in a backwards and consecutive fashion until the sample size was reached. "Overflow" patients were those not included from the original list. See Fig. 1 for reporting of study participation flow [8].

All telephone calls were made by a general surgeon specialized in hernia repair (CP). During the telephone interview, patients were asked whether hernia recurrence had occurred after surgery. Specifically, they were asked about swelling or protrusion in the groin and surgery for recurrence after SPIHR. Patients were also asked whether a wound infection occurred after surgery. The interviewer asked for details to provide Surgical-Site-Infection [9, 10] and Clavien-Dindo classification [11]. Information on surgical site infection during the hospital stay was checked in the patient’s chart.

After discharge, each patient receives a leaflet explaining the usual post-operative course and complications. This is a standard procedure at Shouldice Hospital.

Before the first subject was included, a sample size calculation was conducted to demonstrate the non-inferiority of the polypropylene material. The expected hernia recurrence rate in the polypropylene group was 2%, compared to 1.15% in the wire group. An absolute risk difference of 1% was set as the non-inferiority margin with 1−β = 0.80 and α = 0.05.

Study reporting was conducted in accordance with STROCSS Reporting Guidelines [12].

### Definition of recurrences

Inguinal hernia recurrences were those suspected by the patient (patient's report of bulging and swelling in the groin). Confirmed inguinal hernia recurrences were those noticed in an ultrasound report or clinical examination.

### Definition of surgical site infection

During the phone call the patients were asked, if a surgical site infection occurred after surgery (patient’s report of redness of the wound, pus and fever, necessity of antibiotic intake, reported infection by a physician).

### Material

The choice of surgical material (type/strength) was done by each surgeon independently.

The surgeons at Shouldice Hospital usually use polypropylene in these cases due to the greater mobility and easier handling of this suture in comparison to stainless-steel wire.

### Objective

The primary objective was to determine whether polypropylene is non-inferior to stainless-steel wire suture when used in SPIHR, regarding the recurrence rate reported by patients with a minimum follow-up of 1 year after surgery. The secondary goal was to register the rate of surgical site infections (SSI, I-superficial incisional, II-deep incisional, III-organ space [10]) reported by patients or stated in patients’ charts.

### Inclusion criteria

The study population included male and female patients, aged 16–90 years (the standard age of patients accepted for surgery at Shouldice Hospital), who had elective SPIHR at Shouldice Hospital.

### Exclusion criteria

The exclusion criteria were patients who underwent recurrent repairs, repairs of other hernias (not an inguinal hernia), repairs where mesh or other material other than stainless-steel wire or polypropylene suture was used for repairing the posterior wall of the inguinal canal.

### Univariate analysis

The study groups were compared and described in terms of sociodemographic factors (age, sex, smoking status, body mass index), perioperative characteristics (ASA score, size/location/type of hernia, duration of surgery), and outcome measure (hernia recurrence rate). Categorical variables were presented with frequencies and percentages, while continuous variables were reported with medians and interquartile ranges. Univariate group comparison was conducted using Fisher’s Exact test for categorical variables and Mann–Whitney-U-Tests for continuous variables. All tests were two-tailed, and the significance threshold was set at P < 0.05.

To estimate the strength of the difference the non-directional Cohen’s h was calculated with 95% confidence limits [13].

### Multivariate analysis

Due to differences in baseline characteristics between study groups that may affect the primary outcome, a multivariable analysis was performed. Therefore, a logistic regression model that adjusted for patient (age, sex, BMI, smoking status, AS score) and perioperative features (size/location/type of hernia, duration of surgery, Clavien-Dindo Classification) was conducted. As hernia recurrences are sparse events and may result in (quasi-) separation, classical maximum-likelihood estimation may result in non-convergence issues. Instead, Firth’s penalized maximum likelihood bias reduction estimation procedure was used [14].

Estimates of the marginal means of hernia recurrence rates were calculated using the Emmeans-Package [15]. Weights were applied according to frequencies of factor combinations. Finally, the marginal mean effect of the two materials was compared for non-inferiority (one-sided, α = 0.025, the non-inferior margin of an absolute risk difference of 1%).

For unadjusted analysis of non-inferiority, the difference and 95%-confidence interval of the proportions of hernia recurrence rates between study groups were calculated according to the method of Miettinen and Nurminen [16].

## Results

From the 12 surgeons working at the institution during the study period, four surgeons used solely stainless-steel wire (32 or 34 mm, GerMedUSA^©^), three used solely polypropylene (Prolene^©^ of Ethicon, Inc. or Surgipro^©^ of Medtronic, Inc.; gauge: 2–0 or 0) and five alternated between each type.

### Baseline characteristics and perioperative data

A total of 3583 individuals were screened for eligibility. The consolidated standard of reporting trials flow diagram is depicted in Fig. 1.

A total of 1120 patients, who all received an elective unilateral SPIHR (males: 1057 and females: 63), were interviewed by telephone (Table 1).

**Table 1:** Univariate analysis on baseline characteristics and perioperative data

Variable		SPIHR with stainless steel^1^ (n = 560)	SPIHR with polypropylene^1^ (n = 560)	*p*-value^2^
Age	years	62.0 (49.0, 70.0)	62.0 (52.0, 69.3)	0.8
Sex	male	528 (94.3%)	529 (94.5%)	> 0.9
	female	32 (5.7%)	31 (5.5%)	
Body mass index	kg/m^2^	25.1 (23.5, 26.6)	25.3 (23.7, 26.6)	0.4
ASA-Score	I	140 (25.0%)	143 (25.5%)	0.5
	II	213 (38.0%)	232 (41.4%)	
	III	206 (36.8%)	184 (32.9%)	
	IV	1 (0.2%)	1 (0.2%)	
Smoking status		1 (0.2%)	4 (0.7%)	0.4
Follow-up	months	16.0 (14.0, 16.0)	17.0 (16.0, 19.0)	** < 0.001**
Hernia size	small	62 (11.1%)	65 (11.6%)	** < 0.001**
	medium	356 (63.6%)	231 (41.3%)	
	large	142 (25.4%)	264 (47.1%)	
Hernia type	indirect	325 (58.0%)	308 (55.0%)	0.074
	direct	142 (25.4%)	129 (23.0%)	
	combined	93 (16.6%)	123 (22.0%)	
Hernia side	left	262 (46.8%)	260 (46.4%)	> 0.9
	right	298 (53.2%)	300 (53.6%)	
Length of hospital stay	1 day	1 (0.1%)	0 (0.0%)	**0.004**
	2 days	6 (1.1%)	14 (2.5%)	
	3 days	253 (45.2%)	295 (52.7%)	
	4 days	300 (53.6%)	251 (44.8%)	
Duration of Surgery	minutes	47.0 (40.0, 55.0)	49.0 (42.0, 57.0)	**0.007**

American Society of Anaesthesiologists ASA score

^1^Median (IQR): n (%)

^2^Wilcoxon rank sum test; Fisher’s exact test

A *p*-value below 0.05 has been considered as being statistical significant

Patients in the polypropylene group had larger hernias and a longer duration of surgery. Table 2 depicts the baseline characteristics of the patients according to the use of stainless-steel wire or polypropylene sutures.

**Table 2:** Univariate analysis on primary and secondary objectives

Variable		SPIHR with stainless steel^1^ (n = 560)	SPIHR with polypropylene^1^ (n = 560)	*p*-value^2^
Recurrence rate	Overall	11 (2.0%)	7 (1.3%)	0.5
	Confirmed	*7*	4 (57.1%)	> 0.9
	Not confirmed	*4*	3 (42.9%)	> 0.9
Recurrence was noted	Months	6 (4.5, 6.0)	6 (3.8, 6.0)	0.8
Surgical site infection	None	549 (98.0%)	549 (98.0%)	> *0.9*
	Grade I	9 (1.6%)	8 (1.4%)	
	Grade II	2 (0.4%)	3 (0.5%)	
	Grade III	0 (0.0%)	0 (0.0%)	
Clavien-Dindo-classification	0	547 (98.7%)	548 (98.0%)	*0.9*
	I	3 (0.5%)	4 (0.7%)	
	II	4 (0.7%)	6 (1.1%)	
	III	0 (0.0%)	1 (0.2%)	
	Missing data:	*6*	1	

Confirmed recurrence was confirmed by ultrasound or clinical examination at Shouldice Hospital

^1^Median (IQR): n(%)

^2^Wilcoxon rank sum test; Fisher’s exact test

A *p*-value below 0.05 has been considered as being statistical significant

No significant differences were observed in terms of the recurrence rate.

Results of the univariate analysis on primary and secondary endpoints of the stainless-steel wire and polypropylene populations are shown in Table 3**.** No statistical differences were observed between study groups regarding CDC, rate of SSI and recurrence rate.

**Table 3:** Multivariate analysis on postoperative pain

Variable	exp (Beta)	95% Cl^1^	*p*-value
Gender			
Male	–	–	
Female	3.25	0.59, 12.7	0.2
Age (years)	1	0.96, 1.04	0.9
Body-Mass index	0.91	0.77, 1.12	0.4
Smoking status			
Yes	–	–	
No	0.23	0.02, 32.2	0.4
CDC	1.02	0.01, 190	> 0.9
ASA-Score	0.65	0.29, 1.37	0.3
Duration of Surgery (minutes)	0.98	0.93, 1.03	0.5
Surgical site infection			
None	–	–	
I	3.39	0.00, 7.89	0.7
II	11.6	0.00, 23.234	0.6
Size of hernia			
Small	–	–	
Medium	0.85	0.25, 3.61	0.8
Large	0.71	0.14, 3.77	0.7
Type of hernia			
Indirect	–	–	
Direct	2.47	0.71, 8.72	0.2
Combined	3.17	0.80, 12.4	0.1
Location of hernia			
Left	–	–	
Right	0.29	0.08, 0.86	**0.025**
Suture material			
Stainless steel	–	–	
Polypropylene	0.74	0.24, 2.12	0.6

^1^*CI* confidence interval, *ASA* American society of anaesthesiologists, *CDC* Clavien–Dindo-classification

A *p*-value below 0.05 has been considered as being statistical significant

The unadjusted hernia recurrence rate in the stainless-steel wire group was 2.0% (11 of 560) and in the polypropylene group 1.3% (7 of 560). The difference between the two proportions is 0.71% (95% CI: [− 0.84, 2.38]). As effect size a value of *h* = 0.119 (95% CI: [− 0.005, 0.229]) was obtained.

### Multivariate analysis

**Fig. 2:**
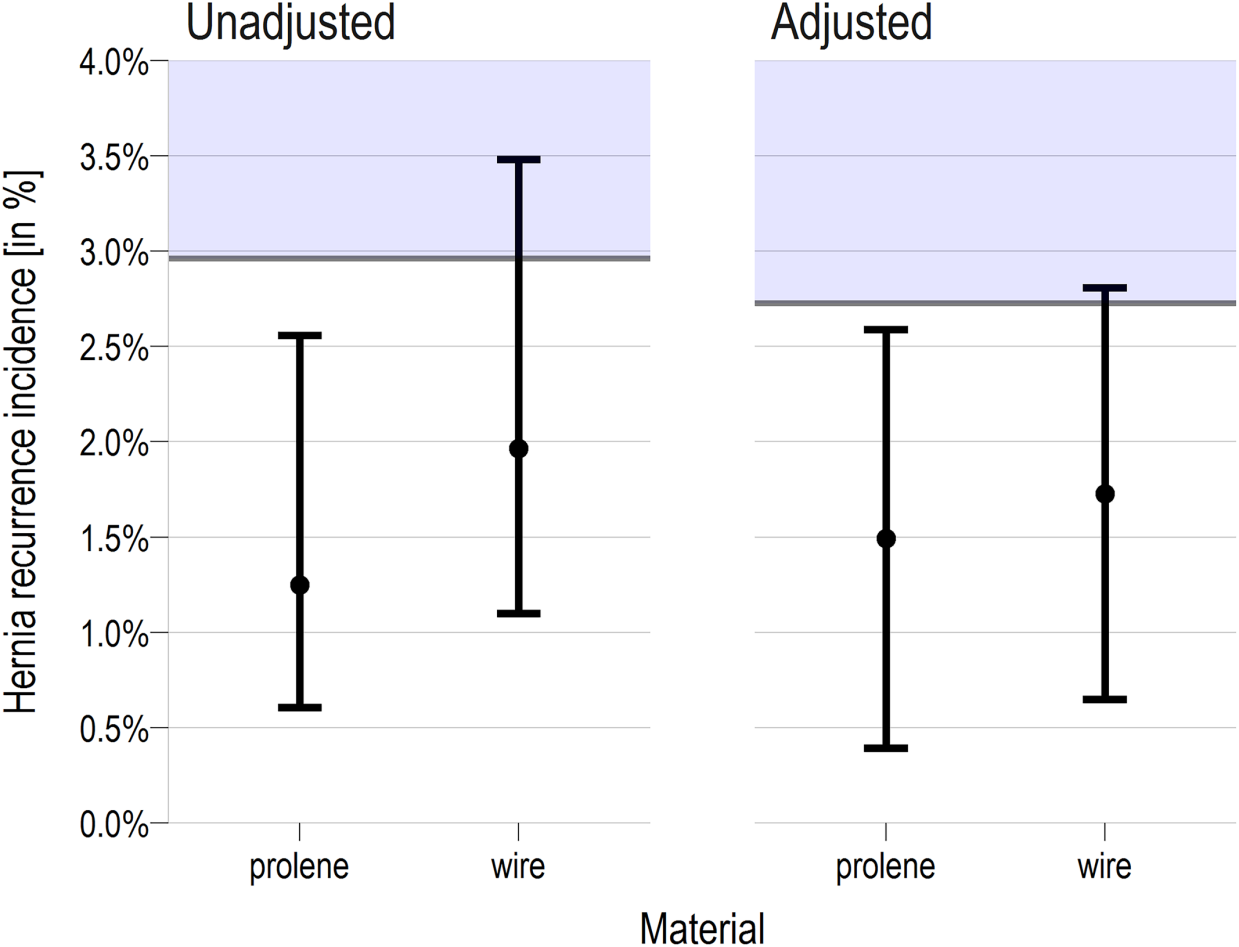
One-sided test on non-inferiority of prolene use in terms of the recurrence rate

Results of the logistic regression to model hernia recurrences are given in Table 4**.** The location side of the hernia showed a significant effect. Based on the results of the logistic regression models, estimated marginal means for the probability of hernia were determined. For the hernia recurrence rate, the estimated proportion was 1.7% (95% CI: [0.5, 2.8]) for stainless steel wire and 1.5% (95% CI: [0.4, 2.6]) for polypropylene material. The one-sided test on non-inferiority yielded a non-significant result (*p* = 0.647) indicating non-inferiority of the polypropylene material. Figure 2 summarizes the results of the analysis.

**Table 4:** Multivariate analysis on the recurrences

Variable	Odds ratio	95% CI^1^	p-value
Age, years	1.00	0.96, 1.04	0.9
Sex			
Male	–	–	
Female	3.25	0.59, 12.7	0.2
Body Mass Index (kg/m^2^)	0.91	0.77, 1.12	0.4
Smoking			
Yes	–	–	
No	0.23	0.02, 32.2	0.4
CDC	1.02	0.01, 190	> 0.9
ASA-Score	0.65	0.29, 1.37	0.3
Duration of Surgery, minutes	0.98	0.93, 1.03	0.5
Surgical Site Infection			
None	–	–	
I	3.39	0.00, 7.89	0.7
II^2^	11.6	0.00, 23.234	0.6
Hernia Side			
Left	–	–	
Right	0.29	0.08, 0.86	0.025
Hernia Type			
Indirect	–	–	
Direct	2.47	0.71, 8.72	0.2
Both	3.17	0.80, 12.4	0.10
Hernia Size			
Small	–	–	
Medium	0.85	0.25, 3.61	0.8
Large	0.71	0.14, 3.77	0.7
Suture Material			
Stainless steel wire	–	–	
Polypropylene	0.74	0.24, 2.12	0.6

*ASA* American society of anaesthesiologists, *CDC* Clavien–Dindo classification

^1^CI represents confidence interval. ^2^no SSI-case in the group with overall recurrences (quasi-complete separation), estimate and standard-errors should not be interpreted

Results of the univariate analysis and the multivariable model on the confirmed hernia recurrence rate are reported in the supplement (Table S1, and Figure S1). A total of 11 recurrence (unconfirmed hernias: n = 7) were confirmed by ultrasound imaging or clinical examination at Shouldice hospital (SPIHR with stainless steel: n = 7; SPIHR with polypropylene: n = 4; p > 0.9).

## Discussion

The classical description of SPIHR involves the use of stainless-steel wire for the reconstruction of the posterior wall in the inguinal canal. This study, in a large population of patients undergoing SPIHR, did not show inferiority for the use of polypropylene sutures instead of stainless-steel wire. This finding may encourage other centers where stainless-steel wire is not easily available to perform a Shouldice Repair in primary inguinal hernias. The findings are in accordance with the recommendations of an international consensus meeting in 2020 [17].

In general, comparing recurrence rates from these different study projects is challenging. The published 1-year recurrence after SPIHR in Europe ranges between 1.2% and 2%. The HerniaMed registry, for example, has diagnosed recurrence data by mailing from patients [18]. The published recurrence rate of 1.15% was reported at Shouldice Hospital when measured by reoperation for hernia recurrence [18–20], where the vast majority of patients underwent SPIHR with the traditionally used stainless-steel wire [1, 20]. Since most hernia surgeons outside Shouldice Hospital use polypropylene as permanent monofilament material when conducting SPIHR the evaluation of this material is of importance. We found a recurrence rate of 1.6% (n = 18) in this study, which is consistent with previous results [18–20].

The guidelines for groin hernias strongly recommend the use of mesh-based repairs for the majority of inguinal hernia patients, except when shared decision-making and/or patients prefer a non-mesh repair [21]. The currently updated guideline states, that in selected groups of patients (Males: EHS-Classification LI, LII, MI; < 40 years of age; Women: EHS-Classification LI, LII, MI, MII) with primary unilateral inguinal hernia repair, SPIHR achieves one-year outcomes comparable to that of Lichtenstein, TEP and TAPP. On the other hand, we found the patient demographics in our study to be comparable to others reported in the literature. The results showed a mean age of 59.70 (± 14.19) years, a body mass index on average of 25.07 (± 2.52) kg/m^2^, the majority of patients were classified as ASA II or III, and 36.2% of patients had a large hernia. Kockerling et al. (2018), reported a mean age of 40 years and a mean BMI value of 24 kg/m^2^ among 2608 individuals, who underwent SPIHR [18]. In comparison, Niebuhr et al. [22], published data on primary unilateral inguinal hernia repair in the TAPP technique among 20,004 individuals from the HerniaMed registry. These patients were not older (on average 56.73 years), not healthier (mainly ASA-II classified) and did not have larger hernias (20% of large inguinal hernias) in comparison to our patients [22]. In addition, Kockerling et al. (2018) published data from 22,111 individuals who underwent primary inguinal hernia Lichtenstein repair in Germany. These patients also had comparable biometric data (mean age 64.6 (± 15.1) years and BMI 25.8 ± 3.6) [19]. Therefore, the indication for SPIHR should potentially be expanded.

The reported recurrence rate is comparable with those from the HerniaMed registry. Niebuhr et al., stated a 1-year recurrence rate of 0.92% following TAPP inguinal hernia repair (n = 20,004) [22]. A total of 0.80% of individuals who underwent Lichtenstein inguinal hernia repair suffered from a recurrence after one year (n = 22,111) [23]. The recurrence rate from the HerniaMed patients (n = 42,115), who underwent laparoscopic or open mesh surgery for inguinal hernia, may be lower due to the follow-up of HerniaMed registry being done by mail and not by phone calls or clinical examinations. Furthermore a large number of hospitals in Germany do not enter data into the HerniaMed registry, which may cause valuable data to be missing from Germany. Several randomized clinical trials on this topic detected a higher incidence of recurrences between one and two years following primary Lichtenstein, TAPP and total extraperitoneal TEP repair [24, 25] . Abbas et al. reported a recurrence rate of approximately 5% when conducting a prospective randomized clinical trial comparing TAPP and Lichtenstein. The mean follow-up was 18 months, but the authors did not state how they diagnosed the recurrences [24]. Anderson et al. (2003) found, by clinical examination, a 12-month recurrence rate of 2.4% after TEP repair [26]. Colak reported a recurrence rate of 2.9% after TEP and 5.9% after Lichtenstein repair. The mean follow-up was 12.04 ± 2.84 months [27]. In addition, Dedemadi detected a 12 month recurrence rate of 4–6% after TAPP, TEP and Shouldice Repair, they didnot state how recurrences were diagnosed [28, 29].

Only 5 patients (0.044%) were active smokers among this study population. This is a lower rate compared to published data from Europe (approximately 11%) [22]. Before surgery at Shouldice Hospital patients are asked to cease or reduce smoking and many patients follow these recommendations. On the other hand, patients at Shouldice Hospital possibly include many former smokers who have similar and persistent smoking-related health problems as patients outside Shouldice Hospital.

Both groups differed in terms of hernia size after conducting a univariate analysis. In the polypropylene group, more patients presented a large hernia. The surgeons at Shouldice Hospital usually use polypropylene in these cases due to the greater mobility and easier handling of this suture in comparison to stainless-steel wire. Also, the polypropylene surgeries were longer, suggesting harder procedures. Some of the newer surgeons at Shouldice Hospital started using polypropylene in all their cases, based on the local impression of similar results [1]. Since the more experienced surgeons were using just stainless-steel wire or polypropylene only in larger hernias, this constitutes a selection bias against polypropylene.

The hernia side appeared to be significantly different, with more hernias repaired on the right side, but with a risk of recurrence higher for hernias on the left side (Table 4). It has been documented that inguinal hernias can be frequently found and operated on the right side [30, 31]. A study found more right-sided recurrent inguinal hernias and reasoned this was due to having a higher prevalence of right-sided primary hernias [31]. The results presented here had similar higher incidence of primary right-sided hernias, but the multivariate analysis revealed left-sided hernias may present as a factor for recurrence. More research is needed on this topic. There is no discussion on the difference in recurrence according to size in the guidelines [21].

The overall reported incidence of SSI was 1.96%. This is similar to other studies and reports in the literature, where SSI incidences range from 0.24 to 5.1% [32–37]. There was no significant difference in the incidence of SSI between the stainless-steel wire and polypropylene groups. This is similar to the findings of Hay et al. (1995) [7], who found that Shouldice Repair with polypropylene compared to stainless-steel wire had similar early and late postoperative complications. It is important to emphasize that it is a reported rate, sometimes the inflammation wound healing phase may cause redness in the wound and be confused with cellulitis, which would trigger diagnosis and possibly treatment. Frequently, there is no agreement between the surgeons and outside physicians in the diagnosis of these mild infections.

The study focused on the comparison of stainless-steel and polypropylene use. Both are non-absorbable suture materials. In the literature also absorbable suture material was investigated. A randomized clinical trial by Hilgert et al. [38] addressed these research question (n = 233). After a minimum follow-up of 24 months after SPIHR the recurrence rate was 5% in the polypropylene and 5% in the polydioxanone (known as PDS) group. The recurrence rate was in general higher than published data. Nevertheless, these findings suggest that the PDS use in SPIHR should be further evaluated in clinical trials [38].

The junction between the wire and the needle is slightly wider than the non-separated prolene material (about 1–2 mm). The wire has to be threaded trough a needle eye. Theoretically, this could result in a buttonhole in the tissue and consecutively weaken the stitched 4-layer posterior wall. This could be an argument in favor of using polypropylene. On the other hand, some surgeons believe that a more robust material such as wire is required because of the risk of damaging the suture material when making the four layers. It is a challenge to scientifically evaluate the impact of these aspects on the outcome.

A still rarely discussed topic in hernia surgery is medical waste and the role of hernia repair in global medical waste. It is well known that laparoscopic surgery leads to a high amount of waste due to the frequent use of disposable materials. The healthcare sector is a significant contributor to greenhouse gas production at 2 gigatons C0_2_ equivalent/year, which corresponds to a global emission of 4.4% [39]. This area is of great importance and resources should be conserved and waste reduced in the face of a global pollution and climate change crisis. The findings of our study demonstrate comparable outcome results to laparoscopic approaches to treat hernias. Hence, there may be a role that non-mesh hernia repair plays in reducing medical waste.

### Limitations

As a limitation of the study, endpoints were determined by telephone calls and review of patients’ charts rather than clinical examination. A further limitation is the short follow-up period of 16.40 (± 1.98) months. The study was conducted unicentric. Although a follow-up examination was performed, a prospective study with stratified randomisation (e.g. hernia size) would have provided more meaningful data. An additional limitation is the fact that we did not analysis data for each of the 12 surgeons.

## Conclusion

The use of polypropylene sutures for the posterior inguinal wall reconstruction is non-inferior to the use of stainless-steel wire regarding recurrence rate at a median follow-up period of 16 months after elective unilateral Shouldice primary inguinal hernia repair. This finding may encourage other centers where stainless-steel wire is not easily available to perform a Shouldice Repair for primary inguinal hernia.

## Supplementary Information

Below is the link to the electronic supplementary material.

Supplementary file1 (PPTX 35 KB)

Supplementary file2 Figure S1 One-sided test on non-inferiority of prolene use in terms of the rate of confirmed recurrences (PNG 161 KB)

## Data Availability

Data is not available for non-commercial researchers.

## References

[1] Mainprize M, Spencer Netto FAC, Degani C, Szasz P (2023) The shouldice method: an expert’s consensus. Hernia 27(1):147–156 35939246 10.1007/s10029-022-02658-y

[2] Shouldice EB (2003) The Shouldice repair for groin hernias. Surg Clin North Am 83(5):1163–1187 14533909 10.1016/S0039-6109(03)00121-X

[3] Shouldice EE (1953) The treatment of hernia. Ontario Med Rev 20:670–684

[4] Shouldice EB (2010) The Shouldice natural tissue repair for inguinal hernia. BJU Int 105(3):428–439 20089101 10.1111/j.1464-410X.2009.09155.x

[5] Usher FC, Allen JE Jr, Crosthwait RW, Cogan JE (1962) Polypropylene monofilament. A new, biologically inert suture for closing contaminated wounds. JAMA 179:780–782 13923961 10.1001/jama.1962.03050100034006b

[6] Brisbane J (1960) A technique for hernia repair. Calif Med 92(5):342–344 13804491 PMC1578025

[7] Hay JM, Boudet MJ, Fingerhut A, Poucher J, Hennet H, Habib E, Veyrières M, Flamant Y (1995) Shouldice inguinal hernia repair in the male adult: the gold standard? A multicenter controlled trial in 1578 patients. Ann Surg 222(6):719–727 8526578 10.1097/00000658-199512000-00005 PMC1235020

[8] Wang F, Schilsky RL, Page D, Califf RM, Cheung K, Wang X, Pang H (2020) Development and validation of a natural language processing tool to generate the CONSORT reporting checklist for randomized clinical trials. JAMA Netw Open 3(10):e2014661 33030549 10.1001/jamanetworkopen.2020.14661 PMC7545295

[9] Berríos-Torres SI, Umscheid CA, Bratzler DW, Leas B, Stone EC, Kelz RR, Reinke CE, Morgan S, Solomkin JS, Mazuski JE, Dellinger EP, Itani KMF, Berbari EF, Segreti J, Parvizi J, Blanchard J, Allen G, Kluytmans JAJW, Donlan R, Schecter WP, Healthcare Infection Control Practices Advisory Committee (2017) Centers for disease control and prevention guideline for the prevention of surgical site infection. JAMA Surg 152(8):784–791 28467526 10.1001/jamasurg.2017.0904

[10] DeBord J, Novitsky Y, Fitzgibbons R, Miserez M, Montgomery A (2018) SSI, SSO, SSE, SSOPI: the elusive language of complications in hernia surgery. Hernia 22(5):737–738 30203373 10.1007/s10029-018-1813-1

[11] Dindo D, Demartines N, Clavien PA (2004) Classification of surgical complications: a new proposal with evaluation in a cohort of 6336 patients and results of a survey. Ann Surg 240(2):205–213 15273542 10.1097/01.sla.0000133083.54934.ae PMC1360123

[12] Mathew G, Agha R, Albrecht J, Goel P, Mukherjee I, Pai P, Dcruz AK, Nixon IJ, Roberto K, Enam SA, Basu S, Muensterer OJ, Giordano S, Pagano D, Machado-Aranda D, Bradley PJ, Bashashati M, Thoma A, Afifi RY, Johnston M, Challacombe B, Ngu JC, Chalkoo M, Raveendran K, Hoffman JR, Kirshtein B, Lau WY, Thorat MA, Miguel D, Beamish AJ, Roy G, Healy D, Ather HM, Raja SG, Mei Z, Manning TG, Kasivisvanathan V, Rivas JG, Coppola R, Ekser B, Karanth VL, Kadioglu H, Valmasoni M, Noureldin A (2021) Strengthening the reporting of cohort, cross-sectional and case-control studies in surgery. Int J Surg 96:106165 34774726 10.1016/j.ijsu.2021.106165

[13] Cohen J (1988) Statistical power analysis for the behavioral sciences, 2nd edn. Lawrence Erlbaum Associates

[14] Firth D (1993) Bias reduction of maximum likelihood estimates. Biometrika 80(1):27–38

[15] Lenth R (2023) Emmeans: estimated marginal means, aka least-squares means. R package version 1.8.8, https://CRAN.R-project.org/package=emmeans. Accessed 23 Oct 2023

[16] Miettinen OS, Nurminen M (1985) Comparative analysis of two rates. Stat Med 4:213–226 4023479 10.1002/sim.4780040211

[17] Lorenz R, Arlt G, Conze J, Fortelny R, Gorjanc J, Koch A, Morrison J, Oprea V, Campanelli G (2021) Shouldice standard 2020: review of the current literature and results of an international consensus meeting. Hernia 25(5):1199–1207 33502639 10.1007/s10029-020-02365-6

[18] Bittner R, Sauerland S, Schmedt CG (2005) Comparison of endoscopic techniques vs Shouldice and other open nonmesh techniques for inguinal hernia repair: a meta-analysis of randomized controlled trials. Surg Endosc 19(5):605–615 15789255 10.1007/s00464-004-9049-9

[19] Köckerling F, Koch A, Adolf D, Keller T, Lorenz R, Fortelny RH, Schug-Pass C (2018) Has shouldice repair in a selected group of patients with inguinal hernia comparable results to lichtenstein, TEP and TAPP techniques? World J Surg 42(7):2001–2010 29299648 10.1007/s00268-017-4433-5 PMC5990577

[20] Malik A, Bell CM, Stukel TA, Urbach DR (2016) Recurrence of inguinal hernias repaired in a large hernia surgical specialty hospital and general hospitals in Ontario. Canada Can J Surg 59(1):19–25 26574701 10.1503/cjs.003915 PMC4734914

[21] Stabilini C, van Veenendaal N, Aasvang E, Agresta F, Aufenacker T, Berrevoet F, Burgmans I, Chen D, de Beaux A, East B, Garcia-Alamino J, Henriksen N, Köckerling F, Kukleta J, Loos M, Lopez-Cano M, Lorenz R, Miserez M, Montgomery A, Morales-Conde S, Simons M (2023) Update of the international HerniaSurge guidelines for groin hernia management. BJS open 7(5):zrad080 37862616 10.1093/bjsopen/zrad080 PMC10588975

[22] Niebuhr H, Wegner F, Hukauf M, Lechner M, Fortelny R, Bittner R, Schug-Pass C, Köckerling F (2018) What are the influencing factors for chronic pain following TAPP inguinal hernia repair: an analysis of 20,004 patients from the herniamed registry. Surg Endosc 32(4):1971–1983. 10.1007/s00464-017-5893-2 29075969 10.1007/s00464-017-5893-2 PMC5845068

[23] Köckerling F, Bittner R, Kofler M, Mayer F, Adolf D, Kuthe A, Weyhe D (2019) Lichtenstein versus total extraperitoneal patch plasty versus transabdominal patch plasty technique for primary unilateral inguinal hernia repair: a registry-based, propensity score-matched comparison of 57,906 patients. Ann Surg 269(2):351–357 28953552 10.1097/SLA.0000000000002541

[24] Abbas AE, Abd Ellatif ME, Noaman N, Negm A, El-Morsy G, Amin M, Moatamed A (2012) Patient-perspective quality of life after laparoscopic and open hernia repair: a controlled randomized trial. Surg Endosc 26(9):2465–2470 22538670 10.1007/s00464-012-2212-9

[25] Scheuermann U, Niebisch S, Lyros O, Jansen-Winkeln B, Gockel I (2017) Transabdominal preperitoneal (TAPP) versus lichtenstein operation for primary inguinal hernia repair—a systematic review and meta-analysis of randomized controlled trials. BMC Surg 17(1):55 28490321 10.1186/s12893-017-0253-7 PMC5424320

[26] Andersson B, Hallen M, Leveau P, Bergenfelz A, Westerdahl J (2003) Laparoscopic extraperitoneal inguinal hernia repair versus open mesh repair: a prospective randomized controlled trial. Surgery 133(5):464–472 12773973 10.1067/msy.2003.98

[27] Colak T, Akca T, Kanik A, Aydin S (2003) Randomized clinical trial comparing laparoscopic totally extraperitoneal approach with open mesh repair in inguinal hernia. Surg Laparosc Endosc Percutan Tech 13(3):191–195 12819504 10.1097/00129689-200306000-00010

[28] Gavriilidis P, Davies RJ, Wheeler J, de Angelis N, Di Saverio S (2019) Total extraperitoneal endoscopic hernioplasty (TEP) versus Lichtenstein hernioplasty: a systematic review by updated traditional and cumulative meta-analysis of randomised-controlled trials. Hernia 23(6):1093–1103 31602585 10.1007/s10029-019-02049-w PMC6938473

[29] Dedemadi G, Sgourakis G, Karaliotas C, Christofides T, Kouraklis G, Karaliotas C (2006) Comparison of laparoscopic and open tension-free repair of recurrent inguinal hernias: a prospective randomized study. Surg Endosc 20(7):1099–1104 16763926 10.1007/s00464-005-0621-8

[30] Nordback I (1984) Side incidence of inguinal hernias. Ann Chir Gynaecol 73(2):87–90 6235767

[31] Perry CP, Echeverri JD (2006) Hernias as a cause of chronic pelvic pain in women. JSLS 10(2):212–215 16882422 PMC3016116

[32] Lee CS, Kim JH, Choi BJ, Lee JI, Lee SC, Lee YS, Oh ST, Kim HJ (2020) Retrospective study on prevalence of recurrent inguinal hernia: a large-scale multi-institutional study. Ann Surg Treat Res 98(1):51–55. 10.4174/astr.2020.98.1.51 31909050 10.4174/astr.2020.98.1.51 PMC6940424

[33] Wang J, Ji G, Yang Z, Xi M, Wu Y, Zhao P, Wang L, Yu W, Wen A (2013) Prospective randomized, double-blind, placebo controlled trial to evaluate infection prevention in adult patients after tension-free inguinal hernia repair. Int J Clin Pharmacol Ther 51(12):924–931 24120711 10.5414/CP201877

[34] Olsen MA, Nickel KB, Wallace AE, Mines D, Fraser VJ, Warren DK (2015) Stratification of surgical site infection by operative factors and comparison of infection rates after hernia repair. Infect Control Hosp Epidemiol 36(3):329–335 25695175 10.1017/ice.2014.44 PMC4683022

[35] HerniaSurge G (2018) International guidelines for groin hernia management. Hernia 22(1):1–165 10.1007/s10029-017-1668-x PMC5809582 29330835

[36] Christou N, Ris F, Naumann D, Robert-Yap J, Mathonnet M, Gillion JF (2022) Risk factors for surgical site infection after groin hernia repair: does the mesh or technique matter? Hernia: J Hernias Abdominal Wall Surg 26(1):233–242 10.1007/s10029-021-02512-7 PMC8881239 34596783

[37] Jaiswal RK, Pandey NK, Tolat A, Kalwaniya DS, Gupta AK, Naga Rohith V, Gurivelli P, Meena R (2023) A Prospective comparative study of laparoscopic totally extraperitoneal (TEP) and laparoscopic transabdominal preperitoneal (TAPP) inguinal hernial repair. Cureus 15(7):e42209 37601986 10.7759/cureus.42209 PMC10439812

[38] Hilgert RE, Dörner A, Wittkugel O (1999) Comparison of polydioxanone (PDS) and polypropylene (Prolene) for Shouldice repair of primary inguinal hernias: a prospective randomised trial. Eur J Surg 165:333–338 10365834 10.1080/110241599750006866

[39] Karliner J, Slotterback S, Boyd R, Ashby B (2019) Health Care's climate footprint. Climate-smart health care series green paper number one and arup.

