# Supplementary material for: Polypropylene vs. stainless-steel wire suture: short-term recurrence rate after shouldice primary inguinal hernia repair, a non-inferior analysis among 1120 patients. A case–control study
Source: Hernia. 2024 Aug 29;28(6):2177–86. doi: 10.1007/s10029-024-03110-z (PMC11530496; doi:10.1007/s10029-024-03110-z)
Supplement: Supplementary file 1 — Supplementary file1 (PPTX 35 KB) [file 10029_2024_3110_MOESM1_ESM.pptx]

## Slide 1
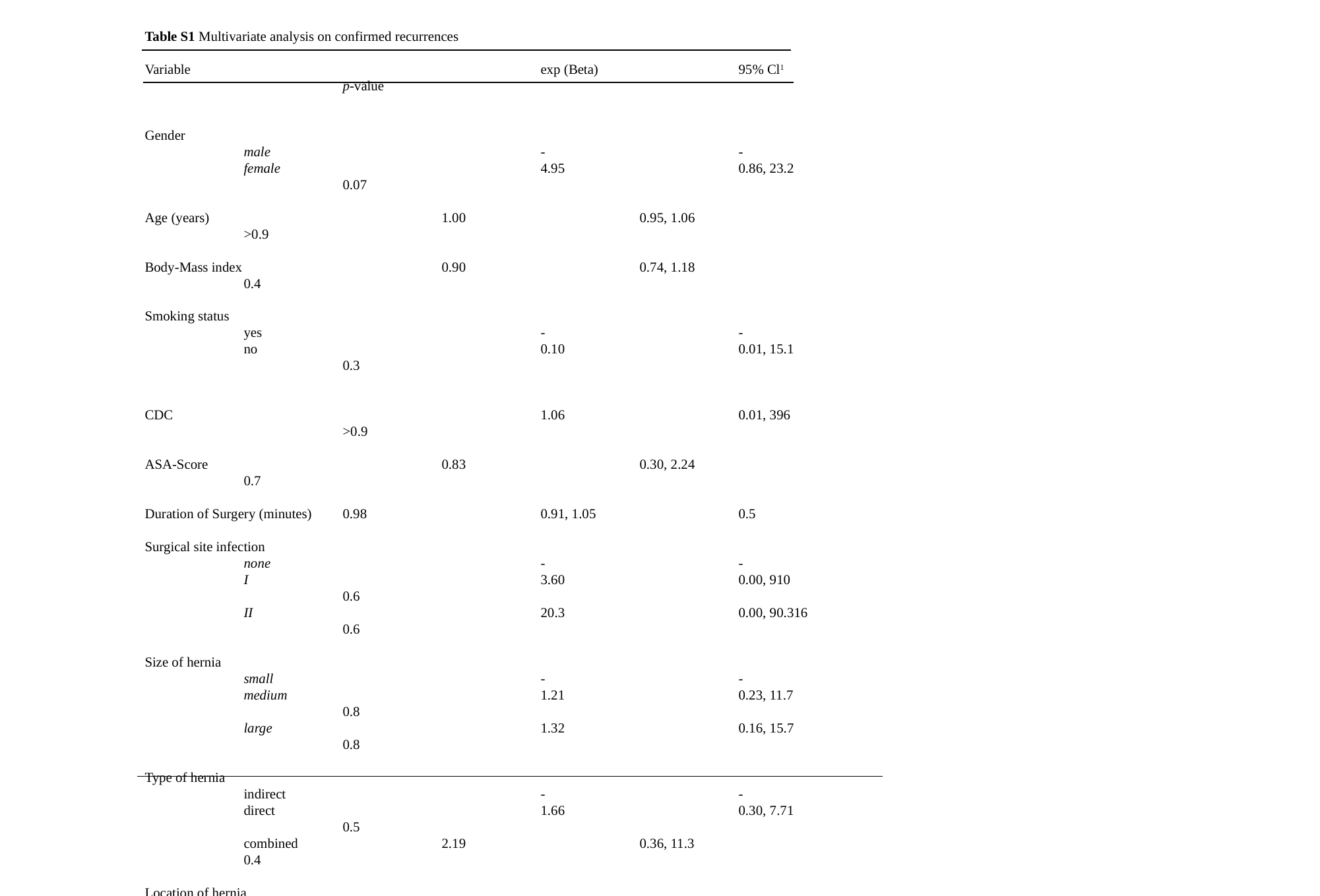

Table S1 Multivariate analysis on confirmed recurrences
Variable				exp (Beta)		95% Cl1		p-value
Gender
	male			-		-
	female			4.95		0.86, 23.2		0.07
Age (years)			1.00		0.95, 1.06		>0.9
Body-Mass index			0.90		0.74, 1.18		0.4
Smoking status
	yes			-		-
	no			0.10		0.01, 15.1		0.3
CDC 				1.06		0.01, 396		>0.9
ASA-Score			0.83		0.30, 2.24		0.7
Duration of Surgery (minutes)	0.98		0.91, 1.05		0.5
Surgical site infection
	none			-		-
	I			3.60		0.00, 910		0.6
	II 			20.3		0.00, 90.316		0.6
Size of hernia
	small			-		-
	medium			1.21		0.23, 11.7		0.8
	large			1.32		0.16, 15.7		0.8
Type of hernia
	indirect			-		-
	direct			1.66		0.30, 7.71		0.5
	combined		2.19		0.36, 11.3		0.4
Location of hernia
	left			-		-
	right			0.21		0.02, 0.89		0.032
Suture material
	stainless steel		-		-
	polypropylene		0.61		0.13, 2.37		0.5
1CI Confidence Interval; ASA American Society of Anaesthesiologists; CDC Clavien-Dindo-Classification
