# Supplementary figures and images for: Polypropylene vs. stainless-steel wire suture: short-term recurrence rate after shouldice primary inguinal hernia repair, a non-inferior analysis among 1120 patients. A case–control study
Source: Hernia. 2024 Aug 29;28(6):2177–86. doi: 10.1007/s10029-024-03110-z (PMC11530496; doi:10.1007/s10029-024-03110-z)

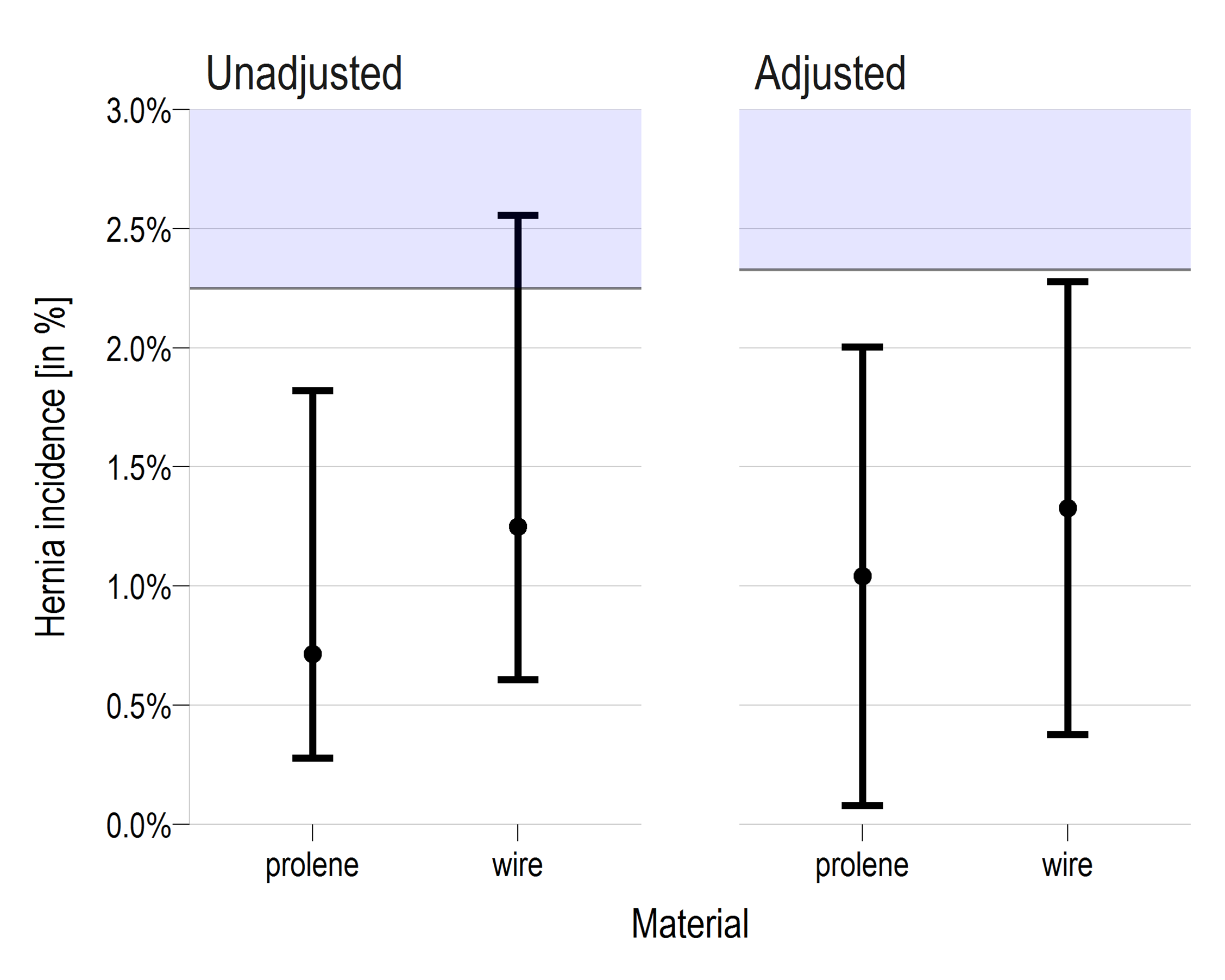

Supplement: Supplementary file 2 — Supplementary file2 Figure S1 One-sided test on non-inferiority of prolene use in terms of the rate of confirmed recurrences (PNG 161 KB) [file 10029_2024_3110_MOESM2_ESM.png]
